# Poor Potential Coverage for 7-Valent Pneumococcal Conjugate Vaccine, Malawi

**DOI:** 10.3201/eid0906.030020

**Published:** 2003-06

**Authors:** Stephen B. Gordon, Stonard Kanyanda, Amanda L. Walsh, Kristy Goddard, Mas Chaponda, Victoria Atkinson, Wakisa Mulwafu, Elizabeth M. Molyneux, Ed E. Zijlstra, Malcolm E. Molyneux, Steve M. Graham

**Affiliations:** * Wellcome Trust Research Laboratories, University of Malawi, Liverpool, U.K.; † University of Malawi College of Medicine, Blantyre, Malawi

**Keywords:** *Streptococcus pneumoniae*, serotypes, serogroups, conjugate vaccine, Africa, Malawi, dispatch

## Abstract

*Streptococcus pneumoniae* infections can be prevented by using new conjugate vaccines, but these vaccines have limited serogroup coverage. We report the first serogrouping data from carried and invasive isolates obtained from children and adults in Malawi. The 7-valent vaccine would cover 41% of invasive isolates from children and 25% from adults. A 9-valent vaccine, including types 1 and 5, would cover 66% of invasive isolates from children and 55% from adults.

*Streptococcus pneumoniae* is the leading cause of bacterial meningitis in both children and adults in Malawi, as well as an important cause of pneumonia, bacteremia, and death for all ages (*1*,*2*). The incidence of *S. pneumoniae* was reduced in the United States and Europe after the licensing of a 7-valent pneumococcal conjugate vaccine (*3*), and the Global Alliance for Vaccines and Immunization (available from: URL: http://www.vaccinealliance.org) has plans to expand the use of the vaccine to sub-Saharan Africa (*4*). However, few data from the central African region exist on which to base a pneumococcal conjugate vaccination program.

## Methods

### Collection of Pneumococcal Isolates

Queen Elizabeth Central Hospital, the main referral hospital for southern Malawi, admits approximately 12,000 children and 10,000 adults per year. From 1996 to 1998, blood cultures and cerebrospinal fluid (CSF) samples were taken from patients admitted to emergency in whom they were indicated. Guidelines suggest that blood cultures should be taken from all febrile patients and lumbar puncture performed if two of three clinical findings (headache, fever, and altered consciousness) indicate meningitis. *S. pneumoniae* were identified from these samples by using colony morphology on blood agar, Optochin sensitivity, and Gram stain. All *S. pneumoniae* isolates collected from these samples were stored in bead and broth bacterial cryopreservers (Prolab Diagnostics, Ontario, Canada) at –80°C for serogrouping at a later date.

### Carriage Study

From March to July 1998, we collected samples from 250 children and 500 adults by using a single posterior nasal swab, with a sterile cotton swab dipped in saline. The swab was then directly rolled on to a sheep blood agar plate and incubated overnight. Colony subcultures with typical morphologic findings were placed on a second blood agar plate with an Optochin disc and stored at 35°C and 5% CO_2_. After 18–24 hours, colonies selected on the basis of typical colony morphologic findings and Optochin sensitivity were stored at –80°C.

### Serogrouping

Stored isolates were recultured for typing by using both sheep blood agar plates and enrichment broth (brain heart infusion and Vitox [Oxoid, Basingstoke, U.K.]). Pneumococcal serogrouping was performed with the Quellung reaction with a standard technique (*5*) and a diagnostic kit from Statens Serum Institut (SSI), Copenhagen, Denmark. This diagnostic kit uses a matrix to group pneumococci covered by the 23-valent pneumococcal vaccine but does not allow typing of pneumococci to the 90 described capsular types. All serogrouping was performed by two investigators (S.B.G. was trained at the World Health Organization [WHO] Pneumococcal Reference Laboratory at SSI, Copenhagen; he trained S.K. who sent a set of serogrouped isolates and isolates that could not be typed by kit to SSI for confirmation of accurate technique).

## Results

A total of 628 invasive pneumococcal isolates were collected from the emergency pediatric and medical service. These isolates consisted of 114 pediatric blood cultures, 206 pediatric CSF samples, 208 adult blood cultures, and 100 adult CSF samples. In the carriage study, 105 isolates were collected from 250 children (42% carriage) and 54 isolates were collected from 500 adults (10.8% carriage). Because of a storage accident, some isolates (predominantly pediatric CSF samples) thawed and were not recovered. The total isolates available for serogrouping was 428 (Figure).

**Figure:**
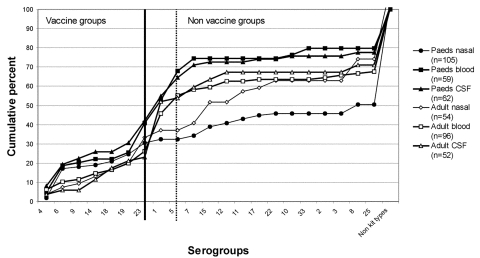
The cumulative percentage of all pneumococcal isolates plotted by source (see key). Serogroups covered by the 7-valent vaccine are plotted to the left of the heavy vertical line, and the potential 9-valent coverage is illustrated by the dotted vertical line.

The serogrouping results are summarized in the Figure. Types 1 and 5 accounted for 27% and 23% of the isolates from pediatric blood and CSF samples, respectively, and 29% and 31% of the isolates from adult blood and CSF samples, respectively. The potential coverage of the 7-valent conjugate vaccine, which excludes these types, was poor. A high percentage of non–23-valent vaccine type pneumococci existed in adult invasive isolates (35%). A sample of 10 such isolates was sent to the WHO Pneumococcal Reference Laboratory for confirmation of serogrouping. Five strains could not be typed or were acapsulate (“rough”) pneumococci, three were not vaccine strains (28F, 16F, and 31), one was *S. oralis,* and one was a misclassified type 7C.

## Discussion

These data are the only recent pneumococcal serogrouping data from children and adults available in this region of Africa. As expected, the data showed high rates of pneumococcal carriage in children and lower rates in adults. The pattern of serogroups showed higher rates of types 1 and 5 than that reported from the United States and Europe. The pattern seen in Malawi is similar to that from West Africa (*6*). A higher incidence of non–23-valent vaccine type pneumococci occurred in our study compared with the incidence in other studies, particularly in our adult isolates; this finding may reflect the high incidence of HIV seropositivity in our population. Recent data from South Africa indicate an increase of typical pediatric carried types (non–23-valent types), causing disease in HIV-infected adults (*7*). Other recent data are available from coastal Kenya (*8*), but both of these datasets represent a different population, in terms of ethnic diversity, geographic separation, and HIV seroprevalence.

The potential coverage of invasive pneumococcal isolates offered by the available 7-valent vaccine was poor in Malawi. Previous estimates have suggested that conjugate vaccine coverage in this region would be 70% to 88% (*9*). We tested a large number of isolates; although some were lost before serogrouping could be performed, the lost isolates probably did not alter the serogroup distribution in our study. In 1980 before the 23-valent polysaccharide vaccine was formulated, serogroups 1 and 5 were considered essential in vaccines for use in Africa (*6*); a 9-valent vaccine, including serogroups 1 and 5, is already under trial in the Gambia and South Africa (*4*). Our study indicates that the inclusion of types 1 and 5 will make a substantial difference to the efficacy of the vaccine in Malawi. Alternatively, protein vaccines may have high efficacy against a still wider range of pneumococci.

Antibiotic use is very low in Malawi compared with other parts of the world, and *Pneumocystis carinii* pneumonia prophylaxis is almost unknown because of the lack of healthcare provision. Very low rates of pneumococcal resistance to penicillin (14%), chloramphenicol (24%), and erythromycin (1%) exist in Malawi (*10*). However, resistance to co-trimoxazole is high (94%) because of the use of sulphamethoxazole-pryrimethamine as firstline anti-malarial drug therapies (*11*). We were not able to compare the incidence of different serotypes between resistant and nonresistant strains because of the small numbers of penicillin-, chloramphenicol-, or erythromycin-resistant isolates and the small number of cotrimoxazole-sensitive isolates.

HIV infection is a particular problem in Malawi, both in adults and children. An estimated 18% of pediatric hospital patients, 72% of adult hospital patients, and 95% of adults with invasive pneumococcal disease are seropositive for HIV in Blantyre, Malawi (*12*). Conjugate vaccine is safe and immunogenic in HIV-infected persons (*13*), and conjugate vaccine was shown to be effective in HIV-infected children, albeit at lower levels than in non–HIV-infected children (*14*). The vaccine coverage of pneumococcal types, vaccine percentage efficacy, and stage of HIV disease in vaccine recipients are critical determinants of whether this strategy will be of value in adults. However, exposure to children carrying pneumococci is a known risk factor for invasive pneumococcal disease in adults (*4*). Therefore, reduced carriage of disease-causing pneumococcal strains in children could reduce the adult incidence of invasive pneumococcal disease in this population. Pneumococcal serogroups will continue to be monitored in disease-causing isolates so that vaccine can be implemented in a timely manner.
